# Risk factors for Gleason score upgrade from prostate biopsy to radical prostatectomy

**DOI:** 10.37349/etat.2024.00259

**Published:** 2024-07-30

**Authors:** Shayan Smani, Vinaik Sundaresan, Soum D. Lokeshwar, Ankur U. Choksi, Jeffrey Carbonella, Joseph Brito, Joseph Renzulli, Preston Sprenkle, Michael S. Leapman

**Affiliations:** Istituto Nazionale Tumori-IRCCS-Fondazione G. Pascale, Italy; ^1^Yale School of Medicine, New Haven, CT 06520, USA; ^2^Department of Urology, Yale School of Medicine, New Haven, CT 06520, USA

**Keywords:** Prostate cancer, Gleason score upgrading, concordance, magnetic resonance imaging fusion biopsy, prostate-specific antigen

## Abstract

Accurate identification of prostate cancer Gleason grade group remains an important component of the initial management of clinically localized disease. However, Gleason score upgrading (GSU) from biopsy to radical prostatectomy can occur in up to a third of patients treated with surgery. Concern for disease undergrading remains a source of diagnostic uncertainty, contributing to both over-treatment of low-risk disease as well as under-treatment of higher-risk prostate cancer. This review examines the published literature concerning risk factors for GSU from time of biopsy to prostatectomy final pathology. Risk factors identified for Gleason upgrading include patient demographic and clinical factors including age, body mass index, race, prostate volume, and biomarker based assays, including prostate-specific antigen (PSA) density, and testosterone values. In addition, prostate magnetic resonance imaging (MRI) findings have also been associated with GSU. Biopsy-specific characteristics associated with GSU include lower number of biopsy cores and lack of targeted methodology, and possibly increasing percent biopsy core positivity. Recognition of risk factors for disease undergrading may prompt confirmatory testing including repeat sampling or imaging. Continued refinements in imaging guided biopsy techniques may also reduce sampling error contributing to undergrading.

## Introduction

Prostate cancer (PCa) is the most commonly diagnosed and second-leading cause of cancer-specific mortality among men in the United States [1]. Patients are screened for PCa with serum prostate-specific antigen (PSA), which offers value in both diagnosis and risk stratification [2]. Recent technological improvements have altered diagnosis and staging strategies for the disease, including multiparametric magnetic resonance imaging (mpMRI), and prostate-specific membrane antigen (PSMA) positron emission tomography (PET) [3, 4]. However, prostate needle biopsy remains the standard diagnostic test [5, 6]. Increasing evidence has supported the use MRI- ultrasound-guided fusion prostate biopsy to improve the accuracy of Gleason score (GS) findings and thus PCa risk stratification [7–9]. Nonetheless, prostate biopsy only samples a small proportion of the prostate and is prone to error. Despite refinements in biopsy technique, patients commonly experience GS discordance and upgrade between biopsy and final pathology on radical prostatectomy (RP). GS upgrading (GSU), defined as an increase in Gleason grade group (GGG) by ≥ 1 or in combined GG by ≥ 1, may occur in as many as 35% of patients who ultimately undergo RP [10]. Modifications to the International Society of Urological Pathology standards for PCa grading have also instituted changes to the reporting of proportions of high-grade disease and tertiary patterns and aggregate reporting of MRI-targeted biopsies to better convey disease risk [11].

The accuracy of GGG assessments from biopsy has assumed greater importance in recent years due to the increasing utilization of conservative treatment options, as well as differences in the intensity and modality of treatment. In particular, disease misclassification at biopsy may cause patients to be inappropriately identified as candidates for conservative management. Such patients who are undergraded are more likely to have adverse pathological features, including extra-prostatic extension and biochemical recurrence [12]. GSU, even after adjusting for known preoperative variables, is a strong predictor of biochemical recurrence after local treatment [13]. Additionally, undergrading of clinically significant PCa could lead to missed escalation of treatment such as combination androgen deprivation therapy-radiation therapy, or omission of pelvic lymph node dissection at the time of RP, further impacting survival. Conversely, overgrading of GSs can lead to overtreatment of less aggressive PCa cases.

Because of its important link to prognosis, researchers have attempted to characterize risk of GSU. Proper identification of patients at risk of GSU could prompt second-level tests such as additional biopsy, prostate MRI, or genomic testing, and may allow for the development of more personalized PCa treatment decisions. The objective of this review is to discuss the risk factors for GSU upon prostatectomy.

## Evidence acquisition

A comprehensive literature search for eligible records was conducted from inception until February 2024. All studies were identified and selected from the PubMed database. We employed a broad search strategy with major search terms that included: “Gleason score”, “upgrade^*^,” “downgrade^*^”, “risk”, “PSA”, “MRI”, “multivariate analysis”, “odds ratio”, etc. which were searched in all fields. The literature search was restricted to the English language and did not include any year restrictions.

Studies were eligible for inclusion if they were (1) original research with experimental design, (2) in the field of urologic oncology, (3) focused on identifying risk factors for GSU, (4) included uni- or multivariate statiscially analysis. Major exclusion criteria included reviews, studies without corresponding full-text such as conference abstracts, ongoing studies, and studies with sample sizes fewer than 50 patients. The final included studies were evaluated in qualitative synthesis. In total, 48 studies were included in this literature review.

## Patient demographics and clinical factors

PCa is a spectrum of heterogenous diseases, some of which are so indolent that they may never impact survival in many patients while others may significantly limit life expectancy. Because of this diversity, there has been a push to detail which demographic factors and other patient clinical factors are valuable in aiding the diagnosis of clinically significant PCa. Among these, increasing age, larger body mass index (BMI), race, and lower prostate volume have often been linked to various clinical end points, including incidence, stage at presentation, biochemical recurrence, and mortality [14, 15]. These risk factors have also been examined as potential risk factors for Gleason upgrading. Table 1 examines identified studies that found significant associations between demographic and clinical factors and GSU.

**Table 1:** Included studies: patient demographic and clinical factors

Authors	Year	Study design	Participants	Study outcomes associated with Gleason upgrading
Leeman et al. [16]	2019	Retrospective cohort	3,571 men with GS6 prostate cancer (PCa)	Older age
Gershman et al. [17]	2013	Retrospective cohort	1,836 men with GS6 on prostate biopsy	Older age, lower prostate weight, and PSA
Epstein et al. [18]	2012	Retrospective cohort	7,643 totally embedded RP and corresponding needle biopsies	Older age, decreasing RP weight, PSA, and increasing maximum percentage cancer/core
Mazzone et al. [19]	2021	Retrospective case-control	424 men with systematic + targeted biopsy and subsequent RP	Age was not associated
Vora et al. [20]	2013	Retrosepective case-control	959 patients with D’Amico low-risk PCa who underwent RP	BMI, African American race, percent of core involved with cancer, increasing CAPRA score, and serum PSA
Freedland et al. [21]	2007	Retrospective cohort	1,113 patients treated with RP from 1996 to 2005 within the Shared Equal Access Regional Cancer Hospital (SEARCH) database who had undergone at least sextant biopsy	Greater PSA, more biopsy cores with cancer, obesity, obtaining less than 8 biopsy cores
Zheng et al. [22]	2023	Retrospective case-control	496 patients who underwent COG-TB and RP	Age, prostate volume, BMI, tumor percentage in biopsy, tumor location
Sundi et al. [26]	2013	Retrospective cohort	1,801 men for met NCCN criteria for very low-risk PCa and underwent RP	African American race, positive surgical margins, and higher CAPRA-S score
Yang et al. [27]	2019	Retrospective cohort	10,089 patients in the NCDB diagnosed from 2010 to 2012 with Gleason 3+4 disease, prostate-specific antigen < 10 mg/mL, and cT1c-2a PCa with < 50% positive biopsy cores	PSA, percentage PBC, age, cT2a versus cT1c, but not black race
Dinh et al. [28]	2015	Retrospective cohort	10,273 patients in the SEER database diagnosed with clinically low risk disease (cT1c/T2a, PSA less than 10 ng/mL, Gleason 3 + 3 = 6) in 2010 to 2011 and treated with prostatectomy	Age, PSA, percent positive cores but not race
Uzzo et al. [30]	1995	Retrospective case-control	1,021 transrectal TRUS-guided sextant pattern prostate biopsies with corresponding RP pathology	Higher prostate gland size
Kulkarni et al. [31]	2006	Retrospective cohort	369 TRUS-guided biopsies of men with PCa with PSA less than 10 ng/mL	Higher TRUS prostate size
Kim et al. [32]	2013	Retrospective case-control	451 patients with PCa with a GS of 6 on biopsy, who underwent RP without neoadjuvant treatment	Smaller prostate volume
Davies et al. [33]	2011	Retrospective case-control	1,251 consecutive patients with D’Amico low risk disease available underwent RP	Smaller prostate volume
Pierorazio et al. [34]	2007	Retrospective case-control	2,600 patients who had undergone RP from 1988 to 2006	Smaller prostate volume, PSA, and age
Freedland et al. [35]	2005	Retrospective cohort	1,602 men treated with RP between 1988 and 2003 at five equal-access medical centers, which composed the SEARCH database	Smaller prostate volume

PSA: prostate-specific antigen; RP: radical prostatectomy; BMI: body mass index; TRUS: transrectal ultrasound; NCCN: National Comprehensive Cancer Network; CAPRA: Cancer of Prostate Risk Assessment; COG-TB: cognitive fusion targeted biopsy; CAPRA-S: CAPRA Post-Surgical scoring system; NCDB: National Cancer Database; PBC: positive biopsy core; SEER: Surveillance, Epidemiology, and End Results

Age has been well described as a risk factor for GSU. In a retrospective analysis of 3,571 men with GG1 disease on transrectal ultrasound (TRUS)-guided biopsy who underwent prostatectomy, Leeman et al. [16] found older age to be a significant predictor of upgrade on surgical pathology (OR 1.05, 95% CI 1.01–1.08, *P* = 0.005). This complements the findings of a similar analysis by Gershman et al. [17] which, in a retrospective analysis included 1,836 patients with GG1 disease who had prostatectomy. The study reported a similar risk of upgrade on prostatectomy per increased year of age (OR 1.05, 95% CI 1.03–1.07, *P* < 0.001) [17]. However, on subgroup analysis of only those patients age > 60, this association is considerably strengthened (OR 2.31, 95% CI 1.50–3.54, *P* < 0.001) [17]. In an analysis of 7,643 paired prostate biopsy and RP specimen recorded in the Institutional Urology Prostate Cancer Database, Epstein et al. [18] found that men who were upgraded at prostatectomy were on average 2 years older. However, despite the large evidence of an age-upgrade association, the magnitude of this effect size was small, limiting the finding’s clinical utility. The introduction of MRI into the PCa diagnostic pathway and further refinements in biopsy technique may reduce lead-time bias and rates of misdiagnosis at initial biopsy with more targeted biopsies. Mazzone et al. [19] examined a cohort of 424 patients receiving systematic and targeted biopsy and found that age was not a significant risk factor for upgrading when MRI-guided biopsy techniques were utilized.

BMI has also been associated with GSU at prostatectomy. Among a sample of 959 urban-residing males, Vora et al. [20] reported a positive association between BMI and GSU (OR 1.04, *P* = 0.02). Freedland et al. [21], examining the Shared Equal Access Regional Cancer Hospital database, found that obese men (BMI ≥ 30 kg/m^2^) had 1.89 times greater odds of GSU when compared to non-obese (BMI < 25 kg/m^2^) (*P* = 0.003). More recently, this association was also seen independent of biopsy technique [22]. Several explanations for this observed association have been offered. First, high BMI may impair proper patient positioning and needle trajectory, making more likely that biopsy insufficiently samples lesions. As such, some studies have suggested that these patients receive more extended biopsy schemes to overcome sampling issues [23]. Additionally, the association between obesity and GSU mirrors the association between obesity and PCa prognosis, likely through similar mechanisms. Obesity is closely related to PCa aggressiveness and PCa-related mortality; several mechanisms have been suggested to underly this including tumor proliferation via the insulin-like growth factor-1 (IGF-1) pathway and oxidative stress-related inflammatory signaling [24, 25].

Race has also been examined as a risk factor for GSU at the time of RP. However, the role of race in GSU is conflicting and with limited reports. Vora et al. [20] used both univariate and multivariate analysis to identify significant predictors of GSU while controlling for clinical parameters. On multivariate analysis, African American men had 1.74 times increased odds of upgrade at the time of prostatectomy. Additionally, a study conducted by Sundi et al. [26] also concluded that African American men diagnosed with very low-risk PCa were more likely to experience upgrade and had worse pathologic outcomes than Caucasian patients (27.3% versus 14.4%, *P* < 0.001). More recently, however, two studies examining the National Cancer Database and Surveillance, Epidemiology, and End Results (SEER) database, each containing samples of more than 10,000 patients, did not observe a significant association between Black versus White race and GSU [27, 28]. These findings were consistent among individuals with GS 3+3 and GS 3+4 cancer at biopsy. As such, it remains unclear what role GSU plays in this relationship. Recent advances in understanding of racial disparities in PCa epidemiology have suggested that much of these differences stem from differential access to care at each level, from screening to diagnosis and treatment [29]. It is possible that in some studies, differences in timeliness and quality of care may contribute to upgrade. Regardless, further research is needed to better characterize this association and its potential causes.

Several studies have examined the influence of prostate size on risk of GSU. An early study by Uzzo et al. [30] suggested that larger glands were associated with disease under-sampling, resulting in an increased risk of GSU. The Prostate Cancer Prevention Trial confirmed these findings among 369 patients with PSA < 10 ng/mL who underwent RP at their institution; TRUS volume was associated with GG4 pattern or greater on biopsy but not at prostatectomy [31]. However, in more recent years, several studies have suggested an inverse relationship—patients with smaller glands are more likely to be upgraded [17, 32–35]. Davies et al. [33] examined 1,251 patients with D’Amico low risk disease and found that men with prostate volumes at the 25th percentile (36 cm^3^) were 50% more likely to experience upgrade than men with prostate volumes at the 75th percentile (58 cm^3^). A later study conducted by Gershman et al. [17] found increased risk for combined GS7 or greater disease with decreasing prostate weight even after controlling for age and PSA among a sample of 1,836 patients with GS6 on initial prostate biopsy. Additionally, Pierorazio et al. [34] found that while smaller volume was associated with upgrading, larger volume was associated with downgrading at prostatectomy.

Several theories have been offered to explain varied findings between small prostates and GSU. In the last decade, advancement in biopsy technology and technique have made possible more targeted and extended biopsy schemes that can improve sampling accuracy in larger glands. Additionally, there is increasing evidence to suggest that PCa found in smaller glands may harbor distinct, more aggressive biology leading to adverse outcomes. One study has suggested that PCa found in the smaller glands are more likely to be androgen-independent, a marker of advanced disease manifestation [35, 36]. Additionally, larger prostates could also serve as physical barriers for the growth of PCa, reducing the risk that tumor extends beyond the gland [37]. However, some studies have suggested that the association between small prostate gland size and adverse clinical outcomes is due to lead-time bias, as improved PSA-based detection is better at identifying PCa in larger prostates, which generally secrete more PSA [38]. However, Freedland et al. [35] point out that this association persists even after exclusion of cT1c biopsy PCa, cases most likely to be subject to lead-time bias. Ultimately, prostate size may affect Gleason upgrading, but more studies are needed to better establish the mechanism underlying this relationship.

### Tumor markers

PSA is a widely used biomarker for the detection and monitoring of PCa. Elevated baseline PSA levels are predictive of advanced PCa diagnosis and future cancer mortality [2, 39]. Despite its diagnostic and prognostic value, the use of PSA as a screening measure has been controversial and has contributed to overtreatment of non-aggressive PCa. Nonetheless, several studies have concluded that increasing PSA before initial biopsy is a significant and independent predictor of GSU [40]. Table 2 identifies these studies in addition to those that examine other tumor marker-based assays. Importantly, the relationship between elevated PSA and GSU remains significant after stratifying for low-risk, favorable-intermediate, and unfavorable intermediate disease found at biopsy [40]. PSA is also an independent predictor of GSU regardless of the number of biopsy cores and technique used to target biopsy (including MRI-fusion biopsy) [41]. Given that this relationship between PSA and GSU persists after controlling for several clinical and demographic factors, PSA continues to serve as a powerful prognostic marker in the contemporary age.

**Table 2:** Included studies: tumor marker-based assays

Authors	Year	Study design	Participants	Study outcomes associated with Gleason upgrading
Mehta et al. [40]	2012	Retrospective case-control	281 cases of GS6 PCa on biopsy with subsequent prostatectomies	PSA, highest percentage cancer at a single biopsy site
Hong et al. [41]	2009	Retrospective case-control	203 patients who underwent radical RRP for low-risk PCa	PSA, number of positive cores
Pham et al. [43]	2020	Retrospective case-control	377 patients with biopsy GS 3+4 who underwent robot-assisted laparoscopic RP from 2014 to 2018	Age, PSA, PSA density (PSAD), PI-RADSv2 score 4–5
Maruyama et al. [44]	2020	Retrospective case-control	403 patients who underwent RP between 2012 and 2018	Increasing PSAD, increasing PI-RADSv2 score
Corcoran et al. [45]	2012	Retrospective case-control	1,516 patients undergoing RP with matching biopsy information	PSAD
Visapää et al. [48]	2010	Retrospective case-control	122 patients with biopsy GS 5 or 6 PCa and a tPSA < 10 ng/mL who underwent RP	Low %fPSA
Kim et al. [50]	2021	Retrospective case-control	300 patients with PSA ≥ 3 ng/mL, PHI and prostate biopsy (71 patients with RP included)	PHI values ≥ 55 and PI-RADS lesions ≥ 4
Gokce et al. [53]	2016	Retrospective case-control	210 low-risk PCa patients eligible for AS, but who underwent RP	Neutrophil-to-lymphocyte ratio ≥ 2.5
Ferro et al. [54]	2019	Retrospective case-control	260 patients who underwent RP and were eligible for AS	Higher Neutrophil-to-lymphocyte, higher platelet-to-lymphocyte, higher monocyte-to-lymphocyte
Pichon et al. [57]	2015	Retrospective case-control	937 patients who were referred to the study center for RP	Serum tesosterone < 3 ng/mL
Lai et al. [46]	2017	Retrospective case-control	67 patients on AS who underwent multiparametric magnetic resonance imaging with MRI/ultrasound (US) fusion–guided biopsy	PSAD, time to biopsy referral, MRI total lesion density, and higher MRI suspicion score score

PCa: prostate cancer; PSA: prostate-specific antigen; tPSA: total PSA; RRP: radical retropubic prostatectomy; PI-RADSv2: Prostate Imaging-Reporting and Data System version 2; %fPSA: percent free PSA; PHI: Prostate Health Index; AS: active surveillance; MRI: magnetic resonance imaging

While PSA is elevated among patients with PCa, PSA is also closely correlated with prostate gland volume. As such, PSA density (PSAD) has emerged as a useful correction factor that can improve diagnostic specificity for clinically significant prostatic disease [42]. PSAD is measured as the ratio of serum PSA concentration to prostate volume measured by TRUS or MRI. A 2020 study of 377 individuals with biopsy GS 3+4 revealed that a PSAD ≥ 0.475 ng/mL^2^ was significantly associated with upgrade at prostatectomy independent of PSA values (*P* < 0.001) [43]. Additionally, Maruyama et al. [44], using both univariate an multivariate analysis, identified 403 patients who underwent prostatectomy at their institution, PSAD was significantly associated with upgrade from GG1 to > GG1 disease (OR 1.10). Corcoran et al. [45] also reported that PSAD was the strongest predictor of GSU between initial biopsy and RP specimen analysis. However, they also found that PSAD lost its predictive ability as prostate volume increased [45]. While many of the studies described here report PSAD based on TRUS findings, Lai et al. [46] report that the relationship between PSAD and upgrading persists among patients who receive MRI/TRUS fusion biopsy.

PCa is also associated with a reduction of the percent of PSA that is unbound in the serum [47]. As a result, percent free PSA (%fPSA) has been clinically incorporated to assess an individual’s cancer risk. Visapää et al. [48] report significant GSU among patients with GS5 or GS6 that had low %fPSA. Recently, the prostate health index has emerged as a commercially available test that combines total PSA, %fPSA, and the p2PSA isoform into a single score with superior specificity for clinically significant or aggressive PCa [49]. While there is limited research exploring its utility to predict GSU, a recent retrospective study analyzed patients with PSA ≥ 3 ng/mL and found that Prostate Health Index (PHI) values ≥ 55 were significant predictors of GSU in RP specimens (OR 3.64, *P* = 0.04) [50].

Neutrophil-to-lymphocyte ratio (NLR) is a marker of progression of cancer-related inflammation among various types of cancer [51]. Lymphocytes are the dominant mediator of inflammation in early tumorigenesis. With increasing stage, neutrophils systemic involvement of disease is correlated with systemic inflammation driven by neutrophilic infiltration, and as such, higher NLR is associated with more advanced disease. In PCa, NLR has been shown to be associated with more aggressive disease [52]. Gokce et al. [53] retrospectively analyzed 210 low-risk PCa patients eligible for AS, who ultimately underwent RP. GSU was significantly more common among patients with an NLR ≥ 2.5 (*P* = 0.04) but had similar rates of upstaging. Ferro et al. [54] later extended these findings to include platelet and eosinophil-to-lymphocyte ratios as predictors of upgrade. Taken together, these tests may play a role in assessing risks of disease undersampling and pathologic upstaging.

PCa development and progression is androgen dependent. Several retrospective and prospective cohort studies have shown an association between low serum total testosterone (TT) and high-grade disease, extraprostatic extension (EPE), and seminal vesicle invasion [55, 56]. Pichon et al. [57] prospectively analyzed 937 individuals who underwent prostatectomy at their institution and found that 20.1% of individuals had GSU while 11.6% were upgraded among normal TT patients (*P* = 0.002). Patients with low TT are subject to more aggressive disease, and more timely definitive treatment is likely warranted in this group to prevent upgrade. Additional studies can help to confirm these early findings and better elucidate the relationship between low TT and GSU among patients without castration-resistant PCa.

### Pre-biopsy imaging

MpMRI has become a central tool in the diagnosis and staging pathway of PCa. Lesions are stratified according to the Prostate Imaging-Reporting and Data System version 2 (PI-RADSv2) scoring system, reflecting probabilities of identifying higher grade PCa. As a result, prostate mpMRI has been evaluated as a staging and prognostic tool that may reduce sampling bias associated with prostate biopsy. Table 3 details studies that examined the relationship of MRI findings and GSU. Park et al. [58] demonstrated that PI-RADSv2-based mpMRI helped identify features associated with aggressive PCa, including GS ≥ 7, larger tumor volume, positive extracapsular extension, and seminal vesicle invasion. Indeed, Brembilla et al. [59] found that mpMRI could identify suitable candidates for extended pelvic node dissection by predicting nodal metastasis. In addition, the role of mpMRI appears to also extend to predicting GSU. Several retrospective analyses spanning multiple institutions have shown that patients with PI-RADS 4 or 5 lesions are significantly more likely to be upgraded on RP [6, 43, 60]. Kamrava et al. [61] show that a PI-RADS 5 lesion is the single most important predictor of GSU (OR 10.56, *P* < 0.01) and that lack of targeted biopsy is an additional predictor of GSU. Song et al. [62] constructed multivariate models based on the results of 443 patients who underwent RP, and found that predictive accuracy of GSU significantly increased with inclusion of PI-RADSv2 score, suggesting its role in risk classification.

**Table 3:** Included studies: pre-biopsy imaging

Authors	Year	Study design	Participants	Study outcomes associated with Gleason upgrading
Pham et al. [43]	2020	Retrospective case-control	377 patients with biopsy GS 3+4 who underwent robot-assisted laparoscopic RP from 2014 to 2018	Older age, elevated PSA, elevated PSAD, PI-RADSv2 score 4–5
Kim et al. [60]	2021	Retrospective case-control	539 patients undergoing RP for biopsy GS 3 + 4 PCa from two tertiary referral centers	Older age, higher PSA, PI-RADS lesion ≥ 4
Song et al. [62]	2018	Retrospective case-control	443 patients who underwent magnetic resonance imaging (MRI) and RP for biopsy-proven GS6 PCa between January 2011 and December 2013	PSAD density > 0.16 ng/mL^2^, number of positive cores ≥ 2, maximum percentage of cancer per core > 20, PIRADSv2 lesion ≥ 4
Kamrava et al. [61]	2015	Retrospective case-control	245 men with a diagnosis of low-risk PCa	PI-RADSv2 5 lesion
Alqahtani et al. [6]	2020	Retrospective case-control	330 men treated with RP between July 2014 and January 2019	Increasing PI-RADSv2 score

RP: radical prostatectomy; PSAD: PSA density; PSA: prostate-specific antigen; PI-RADSv2: Prostate Imaging-Reporting and Data System version 2

### Biopsy-specific factors

Table 4 details studies that identified significant associations between biopsy-based parameters and GSU. As prostate biopsy techniques have been improved over time, the risks of disease under-sampling and misclassification appear to have declined. Several studies prior to the integration of mpMRI and targeted biopsy modalities into the PCa pathway reported that increasing number of biopsy cores improved the accuracy of initial grading [21, 63–66]. Indeed, sextant and low-number biopsy protocols were most often associated with upgrading in these studies [21, 64]. Undersampling may be more pronounced in individuals with larger prostate glands [21]. Thus, patients who receive limited sextant biopsies are more likely to be subject to sampling errors. However, this risk has likely substantially decreased in the contemporary era due to the integration of targeted biopsy and 12-core systematic biopsy schemas which are now standard of care [67].

**Table 4:** Included studies: biopsy-specific factors

Authors	Year	Study design	Participants	Study outcomes associated with Gleason upgrading
Freedland et al. [21]	2007	Retrospective cohort	1,113 patients treated with RP from 1996 to 2005 within the Shared Equal Access Regional Cancer Hospital (SEARCH) database who had undergone at least sextant biopsy	Greater PSA, more biopsy cores with cancer, obesity, obtaining less than 8 biopsy cores
Seisen et al. [63]	2015	Retrospective case-control	1,179 patients managed with RP for a biopsy GS ≤ 6, clinical stage ≤ T2b and preoperative PSA ≤ 20 ng/mL PCa were collected	Length of cancer per core > 5 mm, PSA level > 15 ng/mL, age > 70, number of biopsy cores > 12, and prostate weight > 50 g
San Francisco et al. [64]	2003	Retrospective case-control	466 men diagnosed with localized PCa by needle biopsies who underwent radical retropubic prostatectomy between January 1, 1990 and July 31, 2001	Lower number of biopsies
Ploussard et al. [65]	2009	Retrospective case-control	411 men eligible for AS	12-core biopsy strategy when compared to 21 core scheme
Numao et al. [66]	2007	Retrospective case-control	143 consecutive men in whom PCa was diagnosed by the 3D26 biopsy and who underwent RP	12 or 14 core biopsy when compared to 26 core
Fu et al. [69]	2012	Retrospective case-control	1,632 consecutive men with low-risk PCa who underwent RP between 1993 and 2009	Higher percent tumor involvement
Epstein et al. [18]	2012	Retrospective case-control	7,643 totally embedded RP and corresponding needle biopsies	Older age, decreasing RP weight, PSA, and increasing maximum percentage cancer/core
Vora et al. [20]	2013	Retrospective case-control	959 patients with D’Amico low-risk PCa who underwent RP	BMI, African American race, percent of core involved with cancer, increasing CAPRA score, and serum PSA
Yang et al. [27]	2019	Retrospective cohort	10,089 patients in the NCDB diagnosed from 2010 to 2012 with Gleason 3+4 disease, prostate-specific antigen < 10 mg/mL, and cT1c-2a PCa with < 50% positive biopsy cores	PSA, percentage PBC, age, cT2a versus cT1c, but not black race
Dinh et al. [28]	2015	Retrospective cohort	10,273 patients in the SEER database diagnosed with clinically low risk disease (cT1c/T2a, PSA less than 10 ng/mL, Gleason 3+3=6) in 2010 to 2011 and treated with prostatectomy	Age, PSA, percent positive cores but not race
Hong et al. [41]	2009	Retrospective case-control	203 patients who underwent radical prosatectomy for low-risk PCa	PSA, number of positive cores
Truong et al. [70]	2013	Retrospective case-control	431 patients with Gleason 6 PCa upon biopsy who underwent RP	Higher PSAD, obesity, number of positive cores, and maximum core involvement
Sarici et al. [71]	2014	Retrospective case-control	321 patients who underwent RP for clinically localized PCa at 2 major centers between January 2007 and March 2013	Lower prostate volume, maximum % of cancer in any core, and > 1 core positive for cancer
Athanazio et al. [72]	2017	Retrospective case-control	2,529 patients who underwent biopsy and prostatectomy in our institution from 2005 to 2014	Age ≥ 60 years, PSAD ≥ 0.2, ≥ 2 positive cores, ≥ 5% core tissue involvement
Evans et al. [73]	2016	Retrospective cohort	5,339 cases of RP notified to the Prostate Cancer Outcomes Registry, Victoria, Australia over 6 years (2009–2014) from 46 hospitals	Long interval between biopsy and RP, higher percentage positive biopsy cores
Zhang et al. [74]	2021	Restropective case-control	637 patients who underwent prostate biopsy and RP in our hospital from January 2014 to January 2021	Clinical stage ≥ T2c, the number of positive cores ≥ 3, and positive rate of biopsy

RP: radical prostatectomy; PCa: prostate cancer; PSA: prostate-specific antigen; NCDB: National Cancer Database; CAPRA: Cancer of Prostate Risk Assessment; SEER: Surveillance, Epidemiology, and End Results; PBC: positive biopsy core

Separately, other biopsy metrics such as the percent of cores positive for PCa and percent of biopsy core with tumor involvement have been shown to be associated with biochemical progression in men with organ-confined disease [68, 69]. As such, it is thought that these measures may also be associated with Gleason upgrading. Fu et al. [69] reported that higher percent tumor involvement was a significant independent predictor of GSU among patients with low-risk PCa. These findings have since been confirmed by numerous additional studies [18, 20, 27, 28, 41, 63, 70–72]. The explanation for association between Gleason upgrade and percent tumor involvement on biopsy has been attributed to several factors including tumor diversity, as well as disease sampling. More directly, increased percentage of tumor involvement reflects larger tumor burden within the prostate. Glands which harbor large tumor volume may harbor significant genetic and risk heterogeneity, potentially increasing the chance of initial disease misclassification on biopsy.

In contrast, several studies note a decrease in the risk of GSU with increasing core positivity and tumor involvement. A large retrospective study conducted by Evans et al. [73] showed that when tumor accounted for more than 25% of biopsy volume, the concordance between the biopsy and RP pathological specimens was higher as the percentage increased. Additionally, Zheng et al. [22] evaluated predictors of GSU among patients who underwent cognitive fusion biopsy and found that increasing tumor percentage in biopsy was associated with lower risk of upgrading at prostatectomy. Zhang et al. [74] also found that number of positive cores ≥ 3 was associated with lower risk of upgrading (OR 0.435, *P* = 0.04). These studies cite the use of targeted or extended biopsy modalities which improve the precision and accuracy of each biopsy to effectively reflect the true pathological state of the prostate.

## Impact and future directions

In the modern PSA era, the number of patients diagnosed with low-risk PCa (clinical T1c or T2a or GS ≤ 6), has risen substantially [75]. To limit overtreatment in patients with clinically indolent PCa, less invasive management strategies including active surveillance have been increasingly used to delay definitive management [76]. However, as was most notably discovered by D’Amico et al. [77], roughly 40% of men with GS ≤ 6 at biopsy may have occult, higher-grade disease at the time of prostatectomy. The management and prognosis of PCa is heavily driven by GS. Despite improvement in biopsy approaches, difference between biopsy and postoperative pathology remains an enduring source of uncertainty in the management of localized PCa [78]. Gleason upgrading can lead to unnecessary delays in treatment while downgrading can lead to overtreatment, impacting a patient’s quality of life and healthcare expenditure [69]. As such, understanding factors that underly the discrepancy between biopsy and prostatectomy GS is an area for continued refinement.

Appreciating the possibility of Gleason undegrading contributes to the personalized discussion of PCa decision making. For example, patients with initial “low risk” PCa but with significant risk factors for upgrading including high PSA values and high suspicion MRI scans should be counseled about their risks for disease undersampling. As such, close evaluation including confirmatory testing and repeat prostate biopsy in the setting of risk factors may be warranted.

Improvements in image-guided biopsy modalities can also reduce sampling error that miss higher Gleason grade PCa. Prostate biopsy has traditionally been guided with conventional ultrasound. The use of mpMRI and MRI-TRUS fusion biopsy has led to improvements in the diagnostic assessment for clinically significant PCa, but possibilities of undersampling remain as noted by the presence of GSU on final pathology, especially for PI-RADS 4–5 lesions [79, 80]. Conversely, the addition of mpMRI to the PCa diagnostic pathway has caused risk inflation, often doubling the proportion of National Comprehensive Cancer Network (NCCN) high-risk patients identified at biopsy [81]. In fact, the use of MRI-targeted biopsy has been associated with increased proportions of downgrading on final pathology, possibly due to sampling bias in heterogenous lesions [82].

Because of these limitations, advancements in imaging have been offered and are being evaluated to improve the accuracy of clinical disease sampling. For example, micro-ultrasound (microUS) technology represents a novel technology that may further improve diagnostic accuracy of prostate biopsy. Compared to TRUS with 6–9 Mhz, microUS operates at 29 Mhz and provides a threefold improvement in spatial resolution [83]. Indeed, several studies have shown that the use of microUS alone is non-inferior to MRI-TRUS fusion biopsy for clinically significant Pca (csPCa) detection [84]. Additionally, microUS may also display improved sensitivity while retaining similar specificity when compared to MRI-TRUS [85–88]. Large randomized controlled trials such as the optimization of prostate biopsy–micro-ultrasound versus MRI (OPTIMUM) trial are ongoing to assess the effectiveness of MRI/microUS fusion biopsy [89]. To our knowledge, no study has yet examined rates of GSU in patients biopsied via microUS when compared to other imaging guided techniques.

In addition to advances in imaging, genomic classifiers, such as the Decipher classifier (GenomeDx Biosciences, Vancouver, BC, Canada), have recently been developed as a tissue-based platform to evaluate the expression of 22 genes reflecting pathways involved in cellular proliferation, differentiation, immune modulation, and androgen-receptor signaling [90]. The Decipher classifier has been shown to provide robust predictions of disease outcome among patients with high-risk PCa [90]. However, little is known of Decipher’s ability to estimate the trajectory of untreated low or favorable-risk PCa. One study conducted by Press et al. [91] has shown that elevated Decipher score is associated with short-term biopsy upgrading among patients on active surveillance. Another study by Sheng et al. [92] report that Decipher can independently predict upgrading at prostatectomy. As such, the Decipher genomic classifier may represent a novel approach to predicting GSU and prognostic value in PCa, but larger retrospective studies are necessary to confirm these findings.

## Conclusions

Risk factors for GSU from initial biopsy to prostatectomy have been identified including PSA, PSAD, imaging findings and clinical factors such as age and BMI. Pathologic Gleason upgrading highlights the persistence of sampling error during biopsy, even in the era of image guided prostate biopsy. Identifying patients at higher risk for GSU is an important component of counseling, particularly for patients with favorable risk PCa undergoing initial management with active surveillance, and can inform the need for confirmatory testing.

## Abbreviations

%fPSA: percent free PSA
BMI: body mass index
GS: Gleason score
GSU: Gleason score upgrading
microUS: micro-ultrasound
mpMRI: multiparametric magnetic resonance imaging
MRI: magnetic resonance imaging
NLR: neutrophil-to-lymphocyte ratio
PCa: prostate cancer
PHI: Prostate Health Index
PI-RADSv2: Prostate Imaging-Reporting and Data System version 2
PSA: prostate-specific antigen
PSAD: PSA density
RP: radical prostatectomy
SEARCH: Shared Equal Access Regional Cancer Hospital
SEER: Surveillance, Epidemiology, and End Results;
TRUS: transrectal ultrasound
TT: total testosterone

## Declarations

### Copyright

© The Author(s) 2024.
